# Photorefractive Keratectomy (PRK) is Safe and Effective for Patients with Myopia and Thin Corneas

**Published:** 2016

**Authors:** Mostafa NADERI, Saeed GHADAMGAHI, Khosrow JADIDI

**Affiliations:** 1 Department of Ophthalmology, Baqiyatallah University of Medical Sciences, Tehran, Iran and Bina Eye Hospital, Tehran, Iran

**Keywords:** Photorefractive Keratectomy (PRK), Myopia, Thin Corneas

## Abstract

The aim of this study was to evaluate the long-term safety and efficacy of photorefractive keratectomy (PRK) for patients with myopia and thin corneas. In this retrospective case series, we included 74 eyes of 38 patients with myopia and central corneal thickness (CCT) < 550 µm who underwent PRK and had a mean postoperative follow-up period of four years. The following factors were evaluated: CCT, refraction, uncorrected visual acuity (UCVA), best-corrected visual acuity (BCVA), ablation depth, safety and efficacy indices (i.e., the ratio of the mean postoperative BCVA to the mean preoperative BCVA, and the ratio of the mean postoperative UCVA to mean preoperative the BCVA, respectively), and evidence of corneal ectasia (based on Orbscan topography images).The patients were aged 20 – 46 years (mean ±SD age, 28.18± 6.82 years). The mean ± SD pre- and postoperative CCTwas485.92 ± 9.27 µm and 434.84 ± 20.48 µm, respectively. The mean ± SD pre- and postoperative myopia was -2.77 D ± 1.51 and -0.24 ± 0.39 D, respectively, and the mean ± SD pre- and postoperative astigmatism was -0.82 D ± 0.99 and -0.37 ± 0.37 D, respectively. The mean pre- and postoperative BCVA and postoperative UCVA was 0.011 ± 0.03 Logarithm of the Minimum Angle of Resolution (log MAR), 0.003 ± 0.01 log MAR, and 0.054 ± 0.09 log MAR, respectively. The mean ± SD ablation depth, safety index and efficacy index was 54.34 ± 16.28 µm, 0.02 ± 0.12, and 0.11 ± 0.50, respectively. Regarding the postoperative corneal clarity, 72 eyes (97.3%) had a clear cornea (grade 0) and the remaining two eyes of one patient (2.70%) had a trace haze (grade 1). There was no evidence of corneal ectasia on any of the Orbscan topography images. Thus, among patients with myopia and thin corneas (<500 µm), PRK seems to be acceptable in terms of both safety and efficacy 4 years after surgery, based on the stability of postoperative refraction, visual acuity, and topographic outcomes, and outcomes based on the safety and efficacy indexes.

## Introduction

Central corneal thickness (CCT) is an important preoperative factor that should be measured before conducting refractive surgery using an excimer laser, as it is also a limiting factor when planning for corneal refractive surgery (1-3). Its importance had been demonstrated by multiple studies that have provided evidence of the development of corneal ectasia among patients who have undergone refractive surgery (4-6). Although the minimum CCT that is appropriate for refractive procedures has been considered to be 500 µm (5), previous studies did not prove that refractive procedures on thinner corneas should be contraindicated. Newer studies have provided evidence of the efficacy and safety of surface ablation in subjects with <500-µm-thick corneas, thereby opening up new horizons for the surgical treatment of these patients (7-11). Due to the feasibility and potential cost-effectiveness of photorefractive keratectomy (PRK), we aimed to evaluate its safety and efficacy for the treatment of patients with myopia and thin corneas who were followed up for a mean postoperative period of 4 years.

## MATERIAL AND METHODS

**Study Design and Subjects**

This retrospective case series was approved by the ethics committee of the Baqiyatallah University of the Medical Sciences, Tehran, Iran. The study involved the evaluation of 4500 medical records in 2013 of patients who had been treated with PRK by two surgeons (MN and KhJ) from 2008 to 2010 at the Refractive Surgery Unit of Baqiyatallah Hospital, Tehran, Iran. We included subjects who had either simple or astigmatic myopia with CCT less than 500 µm based on Orbscan topography images and pachymetry using Tomey SP-3000 Pachymeter (Tomey Corp., Japan).

However, we excluded the patients who had previous ocular surgery, suspected keratoconus (based on the Orbscan topography images), a coexisting ocular disease (such as cataracts and glaucoma), or a systemic disease (such as diabetic mellitus and connective tissue disorders). As documented in their medical records, after discussing the possible risks of the operation, informed consent to undergo the surgery was obtained from all the subjects. Based on the data in their medical records and the inclusion and exclusion criteria, we selected 110 of the patients, and contacted them by telephone. Forty patients returned for follow-up assessment, but two were excluded due to the presence of cataracts. The remaining 38 patients signed informed consent forms to take part in the study.

**Assessments**

We recorded the subjects’ preoperative information including age, sex, refraction, CCT, and uncorrected and best-corrected visual acuity (UCVA and BCVA) measured by the LogMAR acuity testing chart, and we recorded the intraoperative ablation depth. We then conducted the following follow-up assessments of the subjects: refraction, CCT, UCVA, BCVA, Orbscan imaging, and a complete ophthalmic examination (including keratometry, fundoscopy, slit-lamp biomicroscopy, and tonometry).

**Surgical Technique**

The PRK was carried out by two surgeons (MN and KhJ). First, under sterile conditions and topical anesthesia (lidocaine 2%), the epithelium was loosened using a 17% alcohol solution for 15 seconds and manually removed using a blunt spatula. The optical zone diameter for all the subjects was 6 mm. Ablation was conducted using a Technolas 217P excimer laser (Technolas Perfect Vision GmbH, Munich, Germany) with a tissue-saving program. A sponge was soaked in mitomycin C (Kyowa Hakko Kogyo, Tokyo) 0.02% and applied to the ablated corneal stroma for a minimum of 10 seconds and a maximum of 35 seconds (depending on the patient’s preoperative refraction), and then the area was vigorously irrigated with 20 ml cold balanced salt solution. Following the surgical procedure, a therapeutic soft contact lens Air Optix; Alcon Laboratories Inc, Fort Worth, Texas, USA) was laid on the eye, and topical antibiotic eyedrop Clobiotic 1%, (Sinadarou, Tehran, Iran) was administered. Postoperatively, the following medications were used: preservative-free artificial tears every 3 hours, Clobiotic 1% eyedrop (Sinadarou, Tehran, Iran) every 4 hours, betamethasone 0.1% eyedrop (Sinadarou, Tehran, Iran) every 6 hours, and oral analgesic drugs. The patients were advised to avoid washing their face for 4 days. Once the epithelial damage healed, the betamethasone 0.1% eyedrop (Sinadarou, Tehran, Iran) was replaced with a fluorometholone eyedrop (Sinadarou, Tehran, Iran), and the dose was tapered off over 2 months. Refraction assessments were repeated 3 months postoperatively.

**Statistical Analysis**

The collected data were entered into SPSS software (version 19; IBM Corp., Armonk, NY, USA). The data are presented as frequencies, percentages, means, standard deviations, and ranges, and, depending on the variable, one of the following statistical tests was applied: t-test, analysis of variance (ANOVA), or chi-square test. In addition, Pearson correlation coefficients were calculated to determine the correlation between postoperative myopia and preoperative myopia, age, CCT, and ablation depth, and between postoperative astigmatism and preoperative astigmatism and age. P < 0.05 was considered statistically significant

## RESULTS

As shown in Table 1, the study included 74 eyes of 38 patients with a mean age of 28.18 ± 6.82 years (range, 20 – 46 years). Of the 74 eyes, 59 (79.7%) belonged to 30 women, and 15 (20.3%) belonged to eight men. The mean follow-up period was 4 years (range, 3 – 5 years), with 23 (31.1%), 33 (44.6%), and 18 (24.30%), eyes being associated with follow-up periods of 3, 4, and 5 years, respectively. As shown in Table 2, the mean ± SD pre- and postoperative simple myopia was -2.77 ± 1.51 D (range, -0.75 – -8.00 D) and -0.24 ± 0.39 D (range, 0.00 – -2.00 D), respectively. The mean ± SD pre- and postoperative astigmatic myopia was -0.82 ± 0.99 D (0.00 – -5.50 D) and -0.37 ± 0.37 D (0.00 – -1.50 D), respectively. The mean ± SD pre- and postoperative CCT was 485.92 ± 9.27 µm (range, 446 – 498 µm) and 434.84 ± 20.48 µm (range, 385 – 477 µm), respectively. The mean ± SD pre- and postoperative BCVA and postoperative UCVA was 0.011 ± 0.03 (range 0.20 – 0.00), 0.003 ± 0.01 (range, 0.10 – 0.00), and 0.054 ± 0.09 (0.40 – 0.00), respectively. Of the 74 eyes assessed, the preoperative BCVA was 0.00 log MAR in 67 eyes (90.5%), 0.10 log MAR in six eyes (8.10%), and 0.20 log MAR in one eye (1.4%); the postoperative BCVA was 0.00 log MAR in 72 eyes (97.3%) and 0.10 log MAR in two eyes (2.7%); and the postoperative UCVA was 0.00 log MAR in 50 eyes (67.6%), 0.10 log MAR in 14 eyes (18.9%), 0.20 log MAR in six eyes (8.1%), 0.3 log MAR in two eyes (2.7%), and 0.4 log MAR in two eyes (2.7%). The mean ± SD ablation depth was 54.34 ± 16.28 µm (range, 29–93 µm). Regarding corneal clarity, 72 eyes (97.3%) had a clear cornea (grade 0) and two eyes of one patient (2.70%) had trace haze (grade 1). The safety index (i.e., the ratio of the mean postoperative BCVA to the mean preoperative BCVA) was 0.02 ± 0.12, and the efficacy index (i.e., the ratio of the mean postoperative UCVA to the mean preoperative BCVA) was 0.11 ± 0.50.

**Table 1:** Baseline demographic variables

**Variable**	**Value**
**Sex**	
Male	15 (20.3)
Female	59 (79.7)
**Age**	28.18 ± 6.82
**Range**	20, 46

SD: standard deviation

Data are presented as No. (%) or Mean ± SD.

**Table 2:** Pre- and postoperative ophthalmologic variables

**Variable**	**Preoperative**		**Postoperative**	
	**Mean ± SD**	**Range**	**Mean ± SD**	**Range**
**Myopia (D)**	-2.77 ± 1.51	0.00, -5.50	-0.24 ± 0.39	0.00, -2.00
**Astigmatism (D)**	-0.82 ± 0.99	-0.75, -8.00	-0.37 ± 0.37	0.00, -1.50
**CCT (µm)**	485.92 ± 9.27	446, 498	434.84 ± 20.48	385, 477
**BCVA (logMAR)**	0.011 ± 0.03	0.20, 0.00	0.003 ± 0.01	0.10, 0.00
**UCVA (logMAR)**	–	–	0.054 ± 0.09	0.40, 0.00
**Myopia (D)**	-2.77 ± 1.51	0.00, -5.50	-0.24 ± 0.39	0.00, -2.00

CCT: central corneal thickness; BCVA: best-corrected visual acuity; UCVA: uncorrected visual acuity; SD: standard deviation

The percentage of patients with preoperative myopia ≤ -3.00 D, -3.1 to -6.00 D, and > -6.00 D was 75.70% (56 eyes), 17.60% (13 eyes), and 6.8% (five eyes), respectively. The percentage of patients with postoperative myopia ≤ -0.50 D and > -0.50 D was 89.10% (66 eyes) and 10.90% (eight eyes), respectively. Regarding changes in postoperative visual acuity, 91.90% (68 eyes) had no change and 8.10% (six eyes) had an improvement of one line on the Snellen chart. Of the six eyes with improved visual acuity, four had postoperative myopia ≤ -3.00 D, one had -3.01 to -6.00 D, and one had > -6.00 D, but the improvements in visual acuity were not significant (p > 0.05). Regarding CCT, 59.5% (44 eyes) had CCT > 430 µm, 35.1% (26 eyes) had CCT 400 – 430 µm, and 5.4% (four eyes) had CCT < 400 µm. None of the postoperative Orbscan topography images showed evidence of corneal ectasia. The Pearson correlation coefficient for age and preoperative myopia was weak and non-significant (0.035; P = 0.76). Regarding the correlations with postoperative myopia, for preoperative myopia, the Pearson correlation coefficient was strong and significant (0.7; P = 0.00), for ablation depth it was moderate and significant (0.46; P = 0.00), and for post-operative CCT it was negative, moderate, and significant (-0.416; P = 0.00). Regarding the correlations with postoperative astigmatism, the Pearson correlation coefficient for age was negative, weak, and significant (-0.315; P = 0.006) and for preoperative astigmatism, it was moderate and significant (0.45; P = 0.000).

## DISCUSSION

This retrospective study showed that PRK was effective, safe, and predictable for the treatment of 74 eyes of 38 myopic patients with <500-µm-thick corneas. There was no evidence of corneal ectasia in any of the subjects. At a mean of 4 years after PRK, 89.10% (66 eyes) had myopia ≤ -0.50 D, which is a higher postoperative percentage than those reported in several previous PRK studies (12-15). This could have occurred because our study had a shorter follow-up period compared to the previous studies or because of the intraoperative use of mitomycin C in our study. However, comparable studies that used a similar follow-up duration with or without the intraoperative use of mitomycin C can help for better clarification.

A recent study by Hashemi et al (9) on the safety and efficacy of PRK in patients with myopia and thin corneas showed that the safety and efficacy indices were 1.01 ± 0.05 and 1.00 ± 0.05, respectively. In addition, the refraction of all the subjects was within ± 0.50 D. In our study; we found that the safety index was 0.02 ± 0.12, the efficacy index was 0.11 ± 0.50, and 89.10% of the subjects had postoperative myopia ≤ -0.50 D. These differences could be due to the difference in the follow-up period, as the study by Hashemi et al had a follow-up period of 1 year while ours had a mean follow-up period of 4 years. One of the limitations of our study is its retrospective nature, as 110 patients were asked to return for ocular examinations (at a mean of 4 years after surgery) and only 40 did so. However, the subjects who did not return for further evaluation may have declined to take part because they were satisfied with their postoperative UCVA. Another limitation of our study compared to the recently published studies in this field is the relatively short follow-up period, as it was much greater (i.e., 10 years) in several similar studies. However, other studies used a similar follow-up period or even shorter periods. Lastly, the lack of a control group of patients with myopia and normal CCT is another limitation of our study. In conclusion, regardless of its limitations, this study showed that PRK is safe and effective for treating patients with myopia and thin corneas. There was no risk of corneal ectasia at a mean of 4 years after surgery. Larger prospective studies with control groups may be necessary to confirm our findings.
